# Epigenetic and Hormonal Modulation in Plant–Plant Growth-Promoting Microorganism Symbiosis for Drought-Resilient Agriculture

**DOI:** 10.3390/ijms242216064

**Published:** 2023-11-08

**Authors:** Cengiz Kaya, Ferhat Uğurlar, Ioannis-Dimosthenis S. Adamakis

**Affiliations:** 1 Soil Science and Plant Nutrition Department, Agriculture Faculty, Harran University, Sanliurfa 63200, Turkey; ferhatugurlar@gmail.com; 2 Section of Botany, Department of Biology, National and Kapodistrian University of Athens, 15784 Athens, Greece; iadamaki@biol.uoa.gr

**Keywords:** plant growth-promoting microorganisms, drought stress, epigenetic modulation

## Abstract

Plant growth-promoting microorganisms (PGPMs) have emerged as valuable allies for enhancing plant growth, health, and productivity across diverse environmental conditions. However, the complex molecular mechanisms governing plant–PGPM symbiosis under the climatic hazard of drought, which is critically challenging global food security, remain largely unknown. This comprehensive review explores the involved molecular interactions that underpin plant–PGPM partnerships during drought stress, thereby offering insights into hormonal regulation and epigenetic modulation. This review explores the challenges and prospects associated with optimizing and deploying PGPMs to promote sustainable agriculture in the face of drought stress. In summary, it offers strategic recommendations to propel research efforts and facilitate the practical implementation of PGPMs, thereby enhancing their efficacy in mitigating drought-detrimental effects in agricultural soils.

## 1. Introduction

Drought stress is a major abiotic factor that impairs plant growth and productivity, and it requires effective strategies to alleviate its impact. Plants have evolved various physiological and molecular mechanisms to cope with drought stress, but these mechanisms often fail when severe conditions exist for prolonged periods of time [1]. Thus, alternative approaches to improve plant drought tolerance and productivity are imperative [2]. One promising possibility involves exploiting the beneficial interactions between plants and PGPMs [3].

PGPMs are a specialized group of microorganisms, including fungi and bacteria, associated with root-modulating gene expression, hormonal regulation, and overall plant physiology [4,5]. By influencing plant physiology, these microorganisms can induce drought tolerance by regulating gene expression and orchestrating hormonal responses [6,7]. Therefore, PGPMs have emerged as key facilitators in enhancing plant resilience to drought [8,9].

Epigenetic modifications, involving enduring alterations in gene expression that do not entail changes to the DNA sequence itself, play a crucial role in enabling plants to adapt to environmental stress, including drought [10]. These complicated molecular adjustments regulate the activation of genes, thereby fine-tuning stress responses. Interestingly, some beneficial microorganisms have been shown to have the capacity to improve plant growth and to fortify drought resilience by shaping the plant’s epigenome. This area of study, although promising, remains relatively unexplored [11,12]. Microbial partnerships and the epigenetic mechanisms that accommodate them offer novel insights into how plants cope with environmental hazards.

However, despite its potential, the field of microbial-induced epigenetic modifications in plants under drought stress remains relatively unexplored, which is mainly due to the limited number of studies existing. Additionally, the complexity of plant–microbe interactions and epigenetic regulation hamper our full understanding of microbial influence on epigenetic responses in plants under drought conditions.

This review advances further from the previous work by Sati et al. [7], who focused on the use of PGPR to mitigate drought stress in agriculture, emphasizing PGPR mechanisms, such as osmotic adjustments and phytohormone production. This review addresses the epigenetic impacts of PGPMs on plants under drought stress, including a wider variety of PGPMs. Further research could be stimulated on this topic and facilitate the development of effective PGPM-based solutions for enhancing crop production in drought-prone agricultural settings.

## 2. Impact of PGPMs on Host Plant Gene Expression under Drought Stress

PGPMs are beneficial microorganisms that enhance plant growth and development through various mechanisms, such as nitrogen fixation, phosphate solubilization, phytohormone production, biocontrol, and stress tolerance [13]. PGPMs include bacteria, fungi, and other microorganisms that can form symbiotic or non-symbiotic associations with plants [14]. Table 1 displays the taxonomic classification of selected PGPMs, specifically those emphasized in this review. 

The investigation into the sophisticated relationship between PGPMs and host plant gene expression under conditions of drought stress has yielded invaluable insights into the mechanisms governing enhanced drought tolerance across diverse plant species and PGPMs. Table 2 summarizes the major results, underlying mechanisms, and genes associated with the mechanisms of microbial contributions to drought tolerance in plants.

More specifically, the revolutionary investigation conducted by Timmusk and Wagner [15] explored priming *Arabidopsis* plants with *Paenibacillus polymyxa*. Their discoveries brought to light an enhanced ability to withstand drought conditions. This enhanced drought tolerance was linked to the activation of specific genes involved in responding to drought stress, including, notably *ERD15* (Early Response to Dehydration 15) and *RAB18* (Late Embryogenesis Abundant 18). Similarly, Liu et al. [16] conducted a study examining the efficacy of another *Paenibacillus polymyxa* strain (strain CR1) in augmenting drought tolerance in *Arabidopsis* and soybean plants. Notably, their research brought attention to the circadian rhythm governing the increased regulation of crucial genes responsive to drought, specifically *RD29A* and *RD29B*, indicating a time-dependent regulatory aspect of these genes in response to drought stress.

In an alternative experimental context, Wang et al. [17] investigated the effects of the so-called BBS group (*Bacillus subtilis* SM21, *Bacillus cereus* AR156, and *Serratia* sp. XY21) in cucumber leaves. Their study revealed that this microorganism group played an essential role in upholding transcriptional levels of vital genes, such as *cAPX* (cytosolic ascorbate peroxidase), *rbcS*, and *rbcL* (RuBisCO small and large subunits). This, in turn, resulted in the strengthening of the antioxidant and photosynthetic machinery of the plants. Consequently, cucumber plants were better equipped to withstand the challenges posed by drought stress due to the enhanced functionality of mentioned crucial biological processes.

In addition, Timmusk et al. [28] found that *Paenibacillus-polymyxa*-produced polyketide-derived metabolites and non-ribosomal peptides (PKs/NRPs) are essential secondary metabolites enhancing drought tolerance. The importance of an A26 Sfp-type 4′-phosphopantetheinyl transferase (*Sfp-type PPTase*) gene was also underlined by their work. It is interesting to note that deactivating this gene increased biofilm production and increased the lifespan of wheat plants facing severe drought stress. This discovery highlights the complex involvement of certain genes and metabolites in the plant’s adaptation to drought stress.

Several studies have examined root colonization by *Pseudomonas chlororaphis* O6, as demonstrated by Cho et al. [18]. They observed that this microbe can induce systemic drought tolerance in *Arabidopsis*. This was achieved through modulating gene expression, which resulted in the activation of genes associated with jasmonic acid (JA) (e.g., *VSP1*, *pdf-1.2*), the salicylic-acid-modulated gene *PR-1*, and the ethylene-responsive gene *HEL*. These findings suggested a multi-hormonal response contributing to enhanced drought resilience, offering compelling prospects for delving deeper into the underlying mechanisms. Similarly, Krishna et al. [19] harnessed a hexa-PGPMs group to enhance drought tolerance in tomato plants. This intervention resulted in the upregulation of various stress-responsive genes, including *DREB*, *APX*, *CAT*, *SOD*, and *P5CS*, further underscoring the pivotal role of PGPMs in enhancing plant resilience to drought.

In the case of the *Pseudomonas simiae* strain AU, Vaishnav and Choudhary [20] emphasized its pivotal role in safeguarding soybean plants by upregulating transcription factors (DREB/EREB), water transporters (PIP and TIP), and osmoprotectants (P5CS, GOLS), thereby effectively promoting drought tolerance. To gain a broader perspective, Kaushal [21] conducted a comprehensive review highlighting the intricate involvement of microbial communities, including arbuscular mycorrhizal fungi (AMF) and plant growth-promoting rhizobacteria (PGPR), in orchestrating a complex network of genes responsible for enhancing drought tolerance. These genes include *ERD15*, *RAB18*, *COX1*, *AP2-EREBP*, *PKDP*, *Hsp20*, *bZIP1*, *COC1*, *LbKT1*, *LbSKOR*, *PtYUC3*, *PtYUC8*, *ADC*, *AIH*, *CPA*, *SPDS*, *SPMS*, *SAMDC*, *14-3-3* genes, *ACO*, *ACS*, jasmonate *MYC2*, *PR1*, *pdf1.2*, *VSP1*, and miRNAs.

Furthermore, Wang et al. [22] delved into the capabilities of *Bacillus amyloliquefaciens* 54 for enhancing drought resistance in tomato plants, noting the upregulation of stress-responsive genes, including *LEA*, *tdi65*, and *ltpg2*. However, the translation of these findings to large-scale agriculture necessitates a deeper understanding of the ecological and economic factors at play. Understanding the potential of *rhizobacteria* for enhancing stress resilience in rice plants, as evidenced by Kakar et al.’s [23] comprehensive study, offers promising insights into agricultural resilience. Nonetheless, addressing practical challenges related to real-world application, scalability, and potential ecological trade-offs is imperative for the responsible and effective deployment of such strategies.

Sarma and Saikia [24] illuminated the elevation of crucial drought-responsive genes, such as *DHN* and *DREB2A*, in mung bean plants subjected to treatment with *Pseudomonas aeruginosa* GGRJ21. This finding presents a promising pathway for improving drought resilience in crops. However, it is imperative to emphasize the need for a comprehensive assessment of the long-term impacts and potential ecological ramifications of such treatments before considering their widespread application.

In an earlier study, Kasim et al. [25] unveiled promising results, showcasing the significant upregulation of stress genes, including ascorbate peroxidase (*APX1*), *S*-adenosyl-methionine synthetase *(SAMS1*), and *HSP17.8*, in the leaves of wheat plants following inoculation with *Azospirillum brasilense* NO40 and *Bacillus amyloliquefaciens* 5113. While Tiwari et al.’s [26] study contributed to our knowledge by highlighting the suppression of key stress-responsive genes, including *DREB1* and *NAC1*, as well as genes associated with ROS scavenging (*CAT*, *APX*, *GST*) and ethylene biosynthesis (*ACO* and *ACS*), in chickpea plants subjected to drought stress following PGPR inoculation, this underscores the complexity of PGPR–plant interactions. To assess the broader implications of these findings, it is crucial to investigate the long-term consequences and ecological considerations associated with such gene modulation strategies.

Lu et al. [27] investigated *Bacillus amyloliquefaciens* FZB42’s role in enhancing drought tolerance in *Arabidopsis*. They found that FZB42 improved plant growth, drought resistance, and defense responses. The study revealed elevated proline levels, increased enzyme activities, and upregulated drought-defense-related genes. Interestingly, FZB42 acted through ethylene (ET) and jasmonate (JA) pathways but not abscisic acid (ABA). An important highlight of the study was the reduced drought resistance observed in a mutant lacking the *epsC* gene, underscoring its critical role in mediating FZB42-induced drought tolerance.

Additionally, Murali et al. [29] isolated ACC deaminase-producing rhizobacteria from pearl millet, including *Bacillus amyloliquefaciens* (MMR04). MMR04 enhanced seed germination and seedling vigor under severe drought stress. Applying MMR04-treated seeds to drought-stressed plants improved growth, chlorophyll content, and water retention. This treatment upregulated antioxidant genes, like *APX1* and *SOD1*, while downregulating ethylene-responsive factor (*ERF-1B*) and drought-responsive genes (*DREB-1E*). These findings highlight the significance of microbe–gene interaction in enhancing drought tolerance through PGPR interventions. 

Similarly, PGPR-inoculated wheat plants exhibited decreased transcript quantities of stress-responsive genes, including *DREB2A* and *CAT1*, compared to untreated plants, further supporting enhanced drought tolerance [30]. Furthermore, *Poncirus trifoliata* seedlings inoculated with AMF (*Glomus mosseae*) showed elevated mRNA levels of genes encoding various antioxidant enzymes, including *CSD1*, *MIOX1*, *GlX1*, and *TTC1*, involved in ROS homeostasis [31]. Rice plants treated with a combination of two PGPR strains, *Brevibacillus laterosporus* B4 and *Bacillus amyloliquefaciens* Bk7, displayed increased expression of stress-related genes. including *TaCTR1* and *TaDREB2*, associated with drought tolerance [23]. In another study, researchers studied the involvement of miRNAs in *Pseudomonas putida* RA-mediated drought tolerance in chickpea plants. This inoculation improved water balance and membrane integrity, key factors for stress resilience. Additionally, the research identified nine miRNAs and their target genes modulated during drought stress, revealing their importance in stress mechanisms. Interestingly, the study found miRNA-target gene expression patterns that opposed each other, whether RA was present or not, unveiling complex regulatory networks in plant responses to drought stress [32].

This comprehensive body of research underscores the complexity and multifaceted nature of the interplay between PGPMs and host plant gene expression in the context of drought stress. Although these results show great promise for improving drought resistance in crops, they also highlight the need for an in-depth understanding of the ecological and practical implications to make sure that these techniques are applied in agricultural operations in a way that is both successful and sustainable.

## 3. Microbial Inoculation and Epigenetic Regulation of Drought-Responsive Genes in Plants

Epigenetic alterations, including DNA methylation and histone modification, have significant roles in plant acclimatization against environmental challenges, such as drought. They result in gene expression alterations, while the underlying DNA sequence remains constant [10]. Microbial–plant interactions can also influence the epigenetic modifications, as some beneficial microorganisms can enhance plant growth and drought tolerance by modulating the plant’s epigenome [33]. 

One of the most prominent epigenetic modifications, DNA methylation, involves the addition of a methyl group to the cytosine bases within DNA strands [34]. This alteration can profoundly affect how genes are expressed by changing how easily transcription factors and chromatin remodeling enzymes can access DNA helixes [35]. However, it is worth noting that the field of microbial-induced epigenetic alterations in plants under drought stress is still relatively unexplored, with just a few investigations shedding light on this fascinating frontier, also underscoring the potential of beneficial microbes to positively influence plant stress responses. It also holds promise for the development of sustainable agricultural practices aimed at mitigating the impact of drought on crops. It also sheds light on the complex interplay between genetic and epigenetic factors in plant stress adaptation.

Although the study of microbial-induced epigenetic changes in drought-stressed plants is still in its early stages, some intriguing investigations have been performed. For example, Da et al. [11] showed that inoculating potato plants with the beneficial bacteria *Burkholderia phytofirmans* (PsJN straian) changed DNA methylation patterns, resulting in drought resistance. Based on their findings, it can be suggested that enhanced DNA methylation plays a role in suppressing PsJN-induced plant growth stimulation. Additionally, gene expression analysis revealed variety-specific responses to PsJN inoculation, highlighting the complex regulatory mechanisms involved. Similarly, Gagné-Bourque et al. [12] showed that *Brachypodium distachyon* Bd21 model grass plants, when inoculated with the endophytic plant growth-promoting bacterium *Bacillus subtilis* B26, had drought-tolerance-enhanced characteristics through the modulation of drought-responsive genes (e.g., *DREB2B-like*, *DHN3-like*, and *LEA-14-A*-*like)* and DNA methylation genes (e.g., *MET1B*-*like*, *CMT3-like*, and *DRM2-like*), which are all involved in drought response.

**Figure 1:**
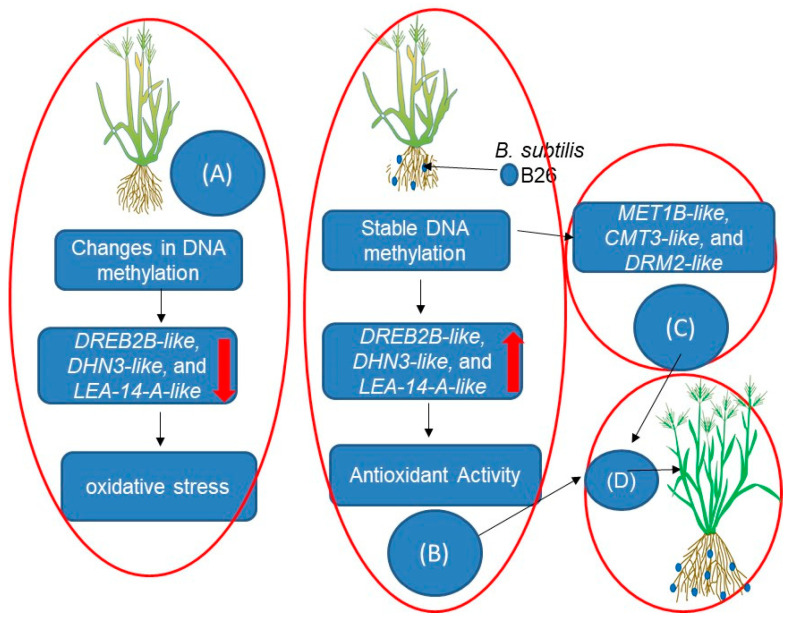
A proposed model of the epigenetic regulation of drought tolerance in the *Bacillus subtilis* B26 plant through microbial colonization. Plants’ DNA methylation patterns may vary in the absence of *Bacillus subtilis* B26 during drought stress (**A**). Transposable element activation and repression of certain genes, such as *DREB2B-like*, *DHN3-like*, and *LEA-14-A-like* genes, can result in increased oxidative stress and poorer drought tolerance. Plants, on the other hand, retain a steady DNA methylation state under drought stress when *B. subtilis* B26 is present (**B**). This stability allows for the correct expression of drought-responsive genes, such as *DREB2B-like*, *DHN3-like*, and *LEA-14-A-like* genes, boosting the plant’s ability to endure drought. The model suggests that microbial influence may extend to epigenetic modifications, including changes in DNA methylation genes (e.g., *MET1B-like*, *CMT3-like*, and *DRM2-like*). These modifications could further impact gene expression and stress responses in plants (**C**), further influencing gene expression and stress responses. Overall, the collective impact of endophytic microbes leads to enhanced drought tolerance (**D**) achieved through the regulation of DNA methylation patterns, gene expression, and potential epigenetic modifications [12]. The red arrows: down for negative regulation, up for positive regulation.

However, the limited number of studies in this domain underscores the need for more comprehensive research to fully grasp the extent of epigenetic regulation by microbes under drought conditions. Figure 1 outlines a model illustrating the epigenetic control of drought tolerance in plants facilitated by *Bacillus subtilis* B26 through microbial colonization. It illustrates how the presence of *B. subtilis* B26 during drought stress promotes stable DNA methylation, thereby positively regulating drought-responsive genes, and potentially influences epigenetic modifications. This microbial influence enhances the plant’s ability to withstand drought conditions, contributing to improved drought tolerance.

In a recent and innovative study conducted by Lephatsi et al. [33], a thorough examination was undertaken to elucidate the metabolic alterations triggered by a microbial-based biostimulant, *Bacillus* group, when applied to maize leaves. The study not only investigated the mechanisms behind growth enhancement and improved drought stress tolerance in maize but also highlighted the transformative potential of microbial-based formulations for inducing significant plant metabolism shifts. Such formulations influence gene expression patterns and DNA methylation profiles, despite being a relatively recent addition to the field of plant–microbe interactions. 

**Figure 2:**
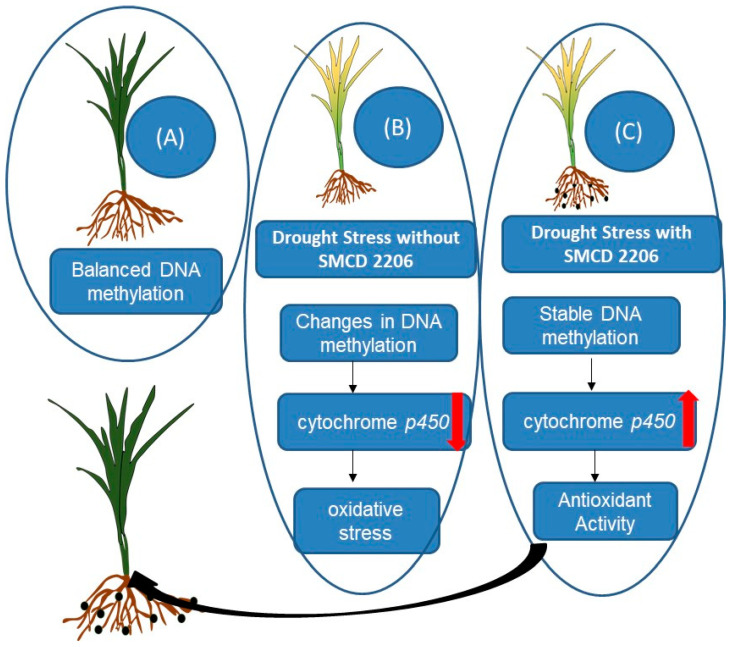
A proposed model of the epigenetic regulation of drought tolerance in wheat by endophytic ascomycete SMCD 2206. (**A**) Under normal conditions, wheat plants have a balanced DNA methylation status and express genes involved in growth and development. (**B**) Under drought stress, wheat plants without SMCD 2206 undergo changes in DNA methylation, leading to the activation of transposable elements and the repression of the cytochrome *p450* gene, which results in increased oxidative stress and reduced drought tolerance. (**C**) Under drought stress, wheat plants with SMCD 2206 maintain a stable DNA methylation status and express genes involved in stress responses. SMCD 2206 reduces the methylation of the cytochrome *p450* gene, which enhances its expression and antioxidant activity. SMCD 2206 also increases the methylation of transposable elements, which prevents their activation and genome instability. SMCD 2206 may also induce other epigenetic modifications, such as histone modifications, that may affect gene expression and stress responses in wheat [36]. The red arrows: up for positive regulation, down for negative regulation. The black arrow indicates “stress response” and “enhanced drought tolerance”.

Hubbard et al. [36] also investigated how drought stress affected wheat and how it interacted with an endophytic ascomyceteSMCD 2206, which is known to increase wheat’s resistance to drought. Using Methyl-sensitive amplified polymorphism (MSAP), they analysed DNA methylation patterns and identified epigenetic modifications induced by SMCD 2206 under drought stress. Their study revealed that drought-stressed wheat plants inoculated with the fungus exhibited DNA methylation patterns similar to unstressed controls, while drought-stressed plants without the endophyte displayed different DNA methylation patterns. Some of these methylation changes were linked to transposable elements and a wheat cytochrome *p450* gene associated with the response to oxidative stress during drought conditions. Figure 2 presents a comprehensive model illustrating the epigenetic regulation of drought tolerance in wheat by the endophytic fungus SMCD 2206. The figure outlines the dynamic changes in DNA methylation, gene expression, and epigenetic modifications under normal and drought stress conditions, shedding light on the pivotal role of SMCD 2206 in enhancing wheat’s resilience to drought. 

Another epigenetic modification in relation to microbial inoculation and drought stress is histone modification, which involves the addition or removal of chemical groups (such as acetyl or methyl groups) to histone proteins [37]. Histone modifications have the potential to influence gene expression by modifying the chromatin structure and regulating the accessibility of the transcriptional machinery [38]. However, as of now, there seem to be no studies exploring the impact of microbial inoculation on histone modifications in plants under drought stress, either by increasing or decreasing the overall histone acetylation or methylation level or by inducing specific histone modification patterns. This represents a fascinating and relatively uncharted area of research with the potential to uncover novel insights into the epigenetic mechanisms governing plant responses to drought.

Table 3 lists the effects of different microorganisms on the epigenetics of plants and how they react to drought stress. The intricate relationships between microorganisms, plants, and epigenetic changes in the context of drought tolerance have been clarified by these investigations. It therefore comes as no so surprise that epigenetic modifications serve as crucial mechanisms for plants to adapt to drought by controlling the expression of stress-responsive genes. Plant–microbial interactions can also influence epigenetic modifications, as some beneficial microorganisms can enhance plant growth and drought tolerance by modulating the plant’s epigenome. The few studies that have been conducted in this advancing field of research demonstrate its enormous potential, while the current studies offer insightful information. Our knowledge of plant–microbe interactions and their applications in sustainable agriculture might be further enhanced by more investigation into the microbial-induced epigenetic changes in plants under drought stress.

## 4. Microbial-Mediated Gene Regulation and Hormonal Response in Plants under Drought Stress 

When plants experience the difficulties of drought stress, microbes have a substantial impact on the control of plant hormones and gene expression. This section gives a summary of current research looking at how microbial inoculation affects particular genes and hormones in plants during drought stress. 

During drought stress, *Pseudomonas mandelii* #29 shows promise in enhancing the uptake of nutrients and the establishment of fungal partnerships within the Mediterranean shrub *Helianthemum almeriense* [39]. It increased the expression of *Terfezia claveryi* AQP (an ectendomycorrhizal symbiont of *Helianthemum* spp. plants) (*TcAQP1*), a gene that encodes a fungal aquaporin. This shows that *P. mandelii* #29 may have a major role in enhancing water transport inside the fungal symbiont, which is an essential adaptation for both the plant and the fungus during times of water shortage. Furthermore, the authors reported an effect on the expression of ABA, a crucial plant hormone strongly associated with drought response mechanisms. These results highlight the capabilities of *P. mandelii* #29 to provide a two-way benefit by promoting favourable fungi relationships and affecting the hormonal responses of the plant, which can be especially helpful for plants in drought conditions. Inoculating corn with *Azospirillum brasilense* SP-7 and *Herbaspirillum seropedicae* Z-152, increased its growth and stress tolerance, according to Curá et al. [40]. These PGPRs were identified as decreasing the expression of *ZmVP14*, a gene involved in ABA production, in maize plants. As a result, the levels of ABA and ethylene, two hormones that increase in response to drought stress, decreased. These findings imply that PGPRs can impact hormonal balance and assist maize plants in coping with drought stress.

*Gluconacetobacter diazotrophicus* PAL5, a diazotroph proficient in nitrogen fixation and root colonization in sugarcane, sparked distinct gene expression responses to drought in sugarcane roots and shoots [41]. This phenomenon suggested a decreased stress level in inoculated plants. Notably, ABA-dependent signaling genes in the inoculated shoots were activated, potentially contributing to enhanced drought resistance. In addition, Vaishnav and Choudhary [20] highlighted the role of the *Pseudomonas simiae* strain AU, a drought-tolerant PGPR, in enhancing soybean plant drought tolerance. They found that *P. simiae* AU inoculation upregulated genes associated with transcription factors dehydration-responsive element binding protein (*DREB*), ethylene-responsive element binding factor (*EREB*), water transporters, plasma membrane intrinsic protein (*PIP*), tonoplast intrinsic protein (*TIP*), and osmoprotectants (P5CS, GOLS) in soybean plants under drought stress. Furthermore, the inoculated plants exhibited increased production of ABA and salicylic acid (SA), along with reduced ethylene emission. 

Manjunatha et al. [4] studied the effects of two endophytic bacteria, *Shewanella putrefaciens* strain MCL-1 and *Cronobacter dublinensis* strain MKS-1, on pearl millet under drought conditions. They found that endophyte-treated plants showed improved growth, root architecture, increased water content, and higher proline accumulation. They also observed elevated levels of ABA and indole acetic acid (IAA) phytohormones, while *C. dublinensis*-inoculated plants showed increased GA content. The study suggests that endophytic bacteria improve pearl millet’s drought tolerance.

**Figure 3:**
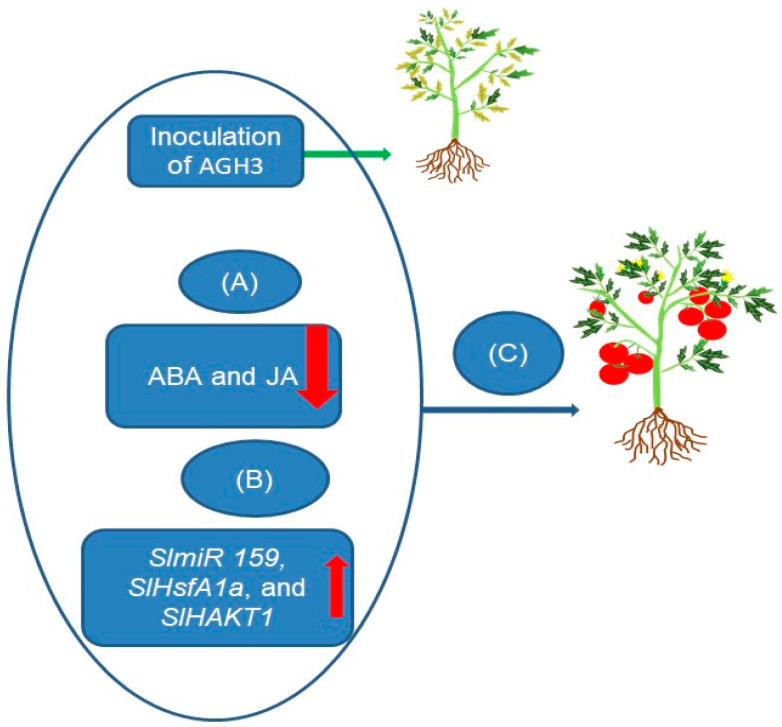
A proposed model of microbial inoculation-mediated genetic and hormonal regulation of drought tolerance in tomato plants. (**A**) Hormonal regulation: during drought stress, the presence of AGH3, a bacteria strain isolated from rhizosphere, leads to a significant reduction in the production of abscisic acid (ABA) and jasmonic acid (JA). This hormonal regulation is depicted by the down arrows. (**B**) Genetic expression: simultaneously, high expressions of key genes, including *SlmiR 159*, *SlHsfA1a*, and *SlHAKT1*, are observed in AGH3-associated tomato plants under drought stress. These gene expressions are represented by the up arrows. (**C**) Enhanced drought tolerance: the combined effect of microbial inoculation, hormonal regulation, and genetic expression leads to enhanced drought tolerance in tomato plants. This enhancement is achieved through the coordinated regulation of hormone production and the expression of genes involved in stress responses [6]. The red arrows: up for positive regulation, down for negative regulation. The green arrow indicates bacterial inoculation of stressed tomato plants. The blue arrow indicates “stress response” and “enhanced drought tolerance” in tomato plants.

In another study, Siraj et al. [6] addressed the challenge of drought stress in crop plants through the utilization of PGPR. They isolated 55 bacterial strains from the rhizosphere of *Achyranthes aspera* L. and *Calotropis procera* (Aiton), with the strains AGH3, AGH5, and AGH9 standing out for their significant production of plant hormones, including also siderophore production and phosphate solubilization. They found that AGH3-associated tomato plants exhibited reduced production of ABA and JA under polyethylene glycol (PEG) stress. The study also revealed increased expression of genes like *SlmiR 159*, *SlHsfA1a*, and *SlHAKT1* in AGH3-associated tomato plants under drought stress. Figure 3 presents an illustrative model depicting how microbial inoculation, specifically AGH3, from the rhizosphere, mediated genetic and hormonal regulation to enhance drought tolerance in tomato plants.

Examining maize plants subjected to concurrent drought and high-temperature stress, Romero-Munar et al. [5] investigated the remarkable regulatory influence of *Rhizophagus irregularis* (AMF) and *Bacillus subtilis*. Their investigation highlighted the cooperative role of these microbial partners in modulating ABA biosynthesis and aquaporin genes, such as *ZmPIP1;3*, *ZmTIP1.1*, *ZmPIP2;2*, and *GintAQPF1*, which are critical for osmotic adjustment in host plants.

Assessing hormone-producing endophytic fungi in soybean plants under drought stress, Bilal et al. [42] co-inoculated soybean with hormone-producing endophytic fungi, *Paecilomyces formosus* LHL10, and *Penicillium funiculosum* LHLO6, enhancing growth, biomass, and photosynthesis while improving nutrient uptake and reducing oxidative stress. The researchers also upregulated key drought-related genes (*GmDREB2*, *GmDREBIB*, *GmERD1*, *GmRD20*, and heat shock proteins). During high-temperature drought (HTD) stress, non-inoculated plants exhibited significantly increased ABA compared to both LHL10- and LHL06-inoculated and co-inoculated plants. Moreover, non-inoculated plants displayed the highest JA production under stress, while LHL06-inoculated plants had the lowest JA levels. This co-inoculation strategy enhances soybean drought resilience in drought-prone regions.

The role of plant microbiomes, particularly those in the rhizosphere, in modulating plant stress, such as drought, was examined also by Lakshmanan et al. [43]. Through the use of ITS2 and 16S rRNA analysis, their work provided techniques for analyzing bacterial and fungi populations. The degradation of the stress-related plant hormone ethylene highlighted that some bacteria can alleviate the effects of drought stress. It was discovered that these bacteria contain the 1-Aminocyclopropane-1-carboxylate (ACC) deaminase enzyme, which inhibits the formation of ethylene and modifies the expression of stress-related genes, therefore improving drought tolerance. The study also presented a high-throughput screening technique for discovering ACC deaminase-producing bacteria capable of successfully reducing plant drought stress. 

The complex relationships between microbial communities, plant gene regulation, hormonal responses, and the reduction of drought stress in many plant species is described in Table 4. These investigations highlight the crucial contributions of microbial interactions, gene expressions, and hormone responses in increasing drought stress resistance in plants. These findings reveal the possibility of utilizing microbial-mediated gene regulation and hormone responses for sustainable crop production, thereby opening the path for creative techniques to increase agricultural output in drought-prone areas. 

## 5. Challenges and Opportunities for PGPM Application in Drought-Stressed Agriculture

Plant–microbe symbioses span a continuum from pathogenic to mutualistic, with functional consequences for both organisms in the symbiosis. To increase sustainable food and fuel production in the future, it is imperative that we harness these symbioses. Plant growth-promoting microbes (PGPMs) offer a promising strategy to enhance plant growth and productivity under drought. However, there are several challenges and opportunities that need to be addressed for the successful application of PGPMs in agriculture.

### 5.1. Challenges

*Environmental variability*: PGPM performance can be inconsistent and variable under different environmental and soil conditions. These microbes may not always colonize plant roots or express their beneficial traits effectively under stressful situations. Therefore, selecting and screening PGPM strains adapted to specific agro-ecological zones and stress scenarios is crucial [44,45].

*Microbial interactions*: PGPMs may interact positively or negatively with other beneficial microorganisms in the plant microbiome, such as mycorrhizal fungi, nitrogen-fixing bacteria, or biocontrol agents. These interactions can affect the efficacy and persistence of PGPMs. Understanding the mechanisms and outcomes of these interactions is essential, and designing compatible and synergistic PGPM groups is necessary [46,47]. 

*Regulation and safety*: Ensuring the safety of PGPM products for commercial use is vital. PGPMs that have not been thoroughly examined or regulated may be hazardous to the environment, animals, or people. Regarding the effectiveness, security, and reliability of PGPM products, compliance with local, state, federal, and international laws and standards is crucial [48].

### 5.2. Opportunities

*Biotechnological integration*: The prospective results from combining PGPMs with biotechnological methods, such as genetic engineering, genome editing, and synthetic biology, are rather encouraging. Researchers can modify or synthesize PGPMs to improve their beneficial characteristics, such as their capacity to produce hormones, withstand stress, and solubilize nutrients. However, plants may be improved or changed to make them more compatible with or sensitive to PGPMs. The innovative and improved PGPM–plant pairs that are more resistant to drought stress may be made with these methods [45].

*Innovative distribution methods*: It is crucial to provide innovative and effective techniques for PGPM application and distribution. In addition to various forms, PGPMs can be given as capsules, granules, pellets, or liquid solutions. They can also be encased in nanoparticles or microgels to improve their viability and durability as well as to protect them from outside threats [49,50]. These methods can improve the shelf-life, transportability, and performance of PGPM products.

*Exploration of diversity*: Exploring new sources and the diversity of PGPMs from different habitats, such as deserts, saline soils, or extreme environments, is a promising choice [51,52]. These unique habitats may harbor novel PGPMs adapted to harsh conditions possessing superior stress tolerance or plant growth promotion traits. Isolating, characterizing, and utilizing these PGPMs can significantly benefit drought-stressed agriculture. 

## 6. Conclusions

The sophisticated relationships between PGPMs and host plant gene expression under drought stress have illuminated critical insights into enhancing drought tolerance across various plant species. Numerous studies have provided concrete evidence of PGPM-induced alterations in gene expression profiles, thereby highlighting specific genes and microorganisms that play pivotal roles in fortifying plants against drought stress. 

These investigations have demonstrated that PGPMs can induce the upregulation of drought-stress-responsive genes, including *ERD15*, *RAB18*, *RD29A*, *RD29B*, and various others, contributing to enhanced drought tolerance. Moreover, the modulation of key genes involved in antioxidant mechanisms, photosynthesis, and osmotic regulation underscores the multifaceted nature of PGPM–plant interactions in mitigating the impacts of drought stress. Epigenetic regulation of drought-responsive genes in plants under microbial influence has emerged as a promising avenue. DNA methylation and histone modifications have been implicated in orchestrating gene expression changes, though research in this area remains relatively unexplored. While initial studies have hinted at the potential for microbial-induced epigenetic modifications, more comprehensive research is required to fully comprehend the extent of this regulation and its implications for plant resilience under drought.

Microbial-mediated gene regulation and hormonal response in plants under drought stress have unveiled how PGPMs can influence hormonal balance, including ABA, ET, and JA, essential players in drought response. These interactions can lead to the upregulation or downregulation of genes associated with stress tolerance and hormone biosynthesis, further emphasizing the pivotal role of PGPMs in enhancing drought tolerance.

## 7. Future Prospects

The research presented here highlights the immense potential of harnessing PGPMs to enhance drought tolerance in crops. However, several aspects for future exploration and development are essential:

*Epigenetic regulation*: The epigenetic modifications induced by PGPMs in plants under drought stress are not fully understood. Elucidating the DNA methylation and histone modification profiles and their roles will provide valuable insights into the regulatory mechanisms at play. Researchers could conduct detailed studies on a specific crop, such as rice, that is highly susceptible to drought stress. For example, they can inoculate rice plants with PGPMs and subject them to drought stress and then perform whole-genome bisulfite sequencing (WGBS) to map DNA methylation changes. This analysis would reveal the precise DNA methylation patterns associated with PGPM-mediated drought tolerance. Further functional genomics studies can investigate the downstream effects of these epigenetic modifications on the expression of key drought-responsive genes.

*Scaling up for agriculture*: Researchers could collaborate with agricultural engineers and industry partners to develop innovative application methods for PGPMs. For instance, they could design specialized PGPM delivery systems that can be integrated into existing irrigation systems. These systems would allow for efficient and large-scale distribution of PGPMs to crops. Simultaneously, they could conduct field trials on a commercial farm to assess the practicality and effectiveness of these systems. The research would not only focus on technical aspects but also consider economic feasibility and ecological impacts, thereby ensuring sustainable large-scale implementation. 

*Long-term effects*: A long-term study could involve establishing dedicated research plots where PGPM-treated crops are cultivated over multiple growing seasons. Researchers could monitor not only crop performance but also changes in soil health and mechanics, microbial communities, and potential ecological consequences. This extended research would provide insights into the persistence of PGPM-induced drought tolerance and its impact on soil ecology over time. 

*Commercial applications*: Collaboration between scientists and industry experts will be crucial in developing PGPM products suitable for commercial use. Research could involve optimizing PGPM production methods to reduce costs, improving formulations for shelf-life stability, and conducting rigorous safety testing. Additionally, researchers could work on developing educational materials and training programs for farmers to ensure the correct and effective application of PGPM products in real-world agricultural settings. 

*Hormonal regulation*: In-depth studies on how PGPMs precisely modulate hormone levels and their downstream effects on plant physiology are warranted. This will enable precise and targeted manipulation of hormonal pathways to enhance drought tolerance.

## Figures and Tables

**Table 1:** Taxonomic classification of selected PGPMs.

PGPMs	Taxa (Domain, Phylum, Class, Order, Family, and Genus)
*Paenibacillus polymyxa*	Bacteria; Bacillota; Bacilli; Bacillales; *Paenibacillaceae*; *Paenibacillus*
*Pseudomonas chlororaphis*	Bacteria; Pseudomonadota; Gammaproteobacteria; Pseudomonadales; *Pseudomonadaceae*; *Pseudomonas*
*Pseudomonas simiae*	Bacteria; Pseudomonadota; Gammaproteobacteria; Pseudomonadales; *Pseudomonadaceae*; *Pseudomonas*
*Bacillus amyloliquefaciens*	Bacteria; Bacillota; Bacilli; Bacillales; *Bacillaceae*; *Bacillus*
*Pseudomonas aeruginosa*	Bacteria; Pseudomonadota; Gammaproteobacteria; Pseudomonadales; *Pseudomonadaceae*; *Pseudomonas*
*Pseudomonas putida*	Bacteria; Pseudomonadota; Gammaproteobacteria; Pseudomonadales; *Pseudomonadaceae*; *Pseudomonas*
*Burkholderia phytofirmans*	Bacteria; Proteobacteria; Betaproteobacteria; Burkholderiales; *Burkholderiaceae*; *Burkholderia*
*Bacillus subtilis*	Bacteria; Bacillota; Bacilli; Bacillales; *Bacillaceae*; *Bacillus*
*Pseudomonas mandelii*	Bacteria; Pseudomonadota; Gammaproteobacteria; Pseudomonadales; *Pseudomonadaceae*; *Pseudomonas*
*Terfezia claveryi*	Eukaryota; Fungi; Ascomycota; Pezizomycetes; Pezizales; *Pezizaceae*; *Terfezia*
*Azospirillum brasilense*	Bacteria; Proteobacteria; Alphaproteobacteria; Rhodospirillales; *Rhodospirillaceae*; *Azospirillum*
*Herbaspirillum seropedicae*	Bacteria; Proteobacteria; Betaproteobacteria; Burkholderiales; *Oxalobacteraceae*; *Herbaspirillum*
*Gluconacetobacter diazotrophicus*	Bacteria; Proteobacteria; Alphaproteobacteria; Rhodospirillales; *Acetobacteraceae*; *Gluconacetobacter*
*Rhizophagus irregularis*	Eukaryota; Fungi; Glomeromycota; Glomeromycetes; Glomerales; *Glomeraceae*; *Rhizophagus*
*Bacillus megaterium*	Bacteria; Bacillota; Bacilli; Bacillales; *Bacillaceae*; *Bacillus*
*Paecilomyces formosus*	Eukaryota; Fungi; Ascomycota; Eurotiomycetes; Eurotiales; *Thermoascaceae*; *Paecilomyces*

**Table 2:** Microbe-induced drought tolerance mechanisms and key genes.

Microbe	Key Findings	Mechanisms	Key Genes Involved	References
*Paenibacillus polymyxa*	Enhanced drought tolerance in *Arabidopsis* plants	Induction of drought stress-responsive genes	*ERD15*, *RAB18*	[15]
*Paenibacillus polymyxa* CR1	Induced drought tolerance in *Arabidopsis* and soybean	Upregulation of critical drought-responsive genes	*RD29A*, *RD29B*	[16]
BBS group	Sustained transcriptional levels of key genes in cucumber leaves	Enhancing antioxidant and photosynthetic machinery	*cAPX*, *rbcS*, *rbcL*	[17]
*Pseudomonas chlororaphis* O6	Induced systemic drought tolerance in *Arabidopsis*	Modulating gene expression	*VSP1*, *pdf-1.2*, *PR-1*, *HEL*	[18]
Hexa-plant growth-promoting microorganism group	Enhanced tomato plant drought tolerance	Up-regulation of stress-responsive genes	*DREB*, *APX*, *CAT*, *SOD*, *P5CS*	[19]
*Pseudomonas simiae* strain AU	Safeguarding soybean plants through modulation of gene expression	Up-regulation of transcription factors, osmoprotectants, and water transporters	*DREB/EREB*, *P5CS*, *GOLS*, *PIP & TIP*	[20]
Microbial-induced systemic tolerance (MIST)	Involvement of microbial communities in gene network orchestration	A complex network of genes including *ERD15*, *RAB18*, *COX1*, and others	*ERD15*, *RAB18*, *COX1*, *PKDP*, *AP2-EREBP*, and more	[21]
*Bacillus amyloliquefaciens* 54	Enhanced drought tolerance in tomato plants	Induction of stress-responsive genes	*lea*, *tdi65*, *ltpg2*	[22]
Rhizobacteria group	Enhanced cold and drought stress tolerance in rice plants	Multiple mechanisms underlying stress tolerance	*CAT1* and *DREB2A*	[23]
*Pseudomonas aeruginosa* GGRJ21	Upregulated expression of drought-responsive genes in mung bean plants	Upregulation of *DREB2A* and *DHN*	*DREB2A*, *DHN*	[24]
*Bacillus amyloliquefaciens* 5113 and *Azospirillum brasilense NO40*	Upregulation of stress genes including *APX1, SAMS1*, and *HSP17.8*	Enhanced drought tolerance of wheat plants	*APX1*, *SAMS1*, *HSP17.8*	[25]
*Pseudomonas putida* MTCC5279	Downregulation of stress-responsive genes including *DREB1*, *NAC1*, and ROS scavenging genes	Downregulation of stress genes	*DREB1*, *NAC1*, *CAT*, *APX*, *GST*	[26]
*Bacillus amyloliquefaciens* FZB42	Improved growth and drought tolerance in *Arabidopsis*	Multiple mechanisms, including ethylene and jasmonate pathways	*RD29A*, *RD17*, *ERD1*, *LEA14*, and more	[27]

**Table 3:** Impact of microbes on plant epigenetics and drought stress responses.

Microbe	Plant Host	Major Findings	Cytosine Methylation Impact	References
*Burkholderia phytofirmans* strain PsJN	Potato varieties: Red Pontiac and Superior	PsJN inoculation caused minimal DNA methylation changes in Red Pontiac, while Superior exhibited increased overall cytosine methylation. Genes displayed variety-specific responses to PsJN.	Enhanced DNA loci methylation observed in Superior, suppressing PsJN-induced growth stimulation.	[11]
*Bacillus subtilis* B26	*Brachypodium distachyon* Bd21	*B. subtilis* B26 increased plant growth, seed yield, and drought tolerance. Upregulated drought-responsive genes and modulation of DNA methylation genes observed.	DNA methylation changes associated with enhanced drought tolerance, involving specific genes (*MET1B-like*, *CMT3-like*, and *DRM2-like*).	[12]
*Bacillus* group (microbial-based biostimulant)	Maize	Biostimulant increased biomass, oxidative stress regulators, and induced metabolic changes in amino acids, phytohormones, flavonoids, and phenolic acids.	Altered metabolic profiles and gene expression patterns, with potential implications for drought resilience.	[33]
Endophytic fungus SMCD 2206	Wheat	SMCD 2206 colonization resulted in similar DNA methylation patterns to unstressed controls in drought-stressed wheat seedlings. Distinct DNA methylation patterns in endophyte-free, drought-stressed plants.	Epigenetic changes associated with SMCD 2206 colonization, with implications for drought resistance.	[36]

**Table 4:** Microbial-mediated gene regulation, hormonal responses, and drought mitigation in plants.

Microbes	Genes Studied	Hormones Investigated	Drought-Related Findings	References
*Pseudomonas mandelii* #29, *Terfezia claveryi*,	*T. claveryi* AQP (*TcAQP1*), Microtubule-associated	ABA	*P. mandelii* #29 enhanced fungal colonization and nutrient uptake under drought stress. Upregulated genes included *TcAQP1*, Microtubule-associated protein, and Predicted 3′−5′ exonuclease. Tripartite interactions improved plant resilience.	[39]
*Azospirillum brasilense* SP-7, *Herbaspirillum* *seropedicae* Z-152	*ZmVP14* and, Other genes	ABA, ET	PGPR-inoculated plants showed increased drought tolerance, higher biomass, reduced ABA and ET, and improved osmoregulation.	[40]
*Gluconacetobacter* *diazotrophicus* PAL5	Multiple genes involved in hormone pathways	ABA and ET	Inoculated plants exhibited increased drought tolerance, unique gene expression in roots, and ABA-dependent signaling in shoots.	[41]
*Pseudomonas simiae* strain AU	Transcription factors (*DREB/EREB*), Osmoprotectants	ABA, SA and ET	Upregulation of drought-related genes and hormone pathways, increased proline and sugar levels.	[20]
55 bacterial strains (*AGH3*, *AGH5*, *AGH9*)	*SlmiR 159*, *SlHsfA1a*, *SlHAKT1*	ABA, JA	Improved growth, reduced ABA and JA production, and increased gene expression under drought stress.	[6]
*Rhizophagus irregularis* (AM), *Bacillus* *megaterium* (Bm)	*ZmPIP1;3*, *ZmTIP1.1*, *ZmPIP2;2*, *GintAQPF1*	ABA, JA, SA, IAA	Dual inoculation mitigated drought and high-temperature stress, improving photosynthesis, root hydraulic conductivity, and regulating aquaporin genes and plant sap hormones.	[5]
*Shewanella putrefaciens* MCL-1, *Cronobacter* *dublinensis* MKS-1	*SbNCED*, *SbGA20oX*, *SbYUC*, *SbAP2*, *SbSNAC1*, *PgDREB2A*	ABA, IAA, GA	Endophyte-inoculated plants exhibited improved growth, higher hormone levels, and upregulated genes associated with phytohormone biosynthesis and drought-responsive transcription factors.	[4]
*Paecilomyces formosus* LHL10, *Penicillium* *funiculosum* LHLO6	Drought-related genes (*GmDREB2*, *GmDREB1B*, *GmERD1*, *GmRD20*)	Endogenous ABA and JA	Co-inoculation improved soybean growth, photosynthetic activity, antioxidant enzyme activities, nutrient uptake, and reduced oxidative damage under drought stress.	[42]
Various plant-associated microbiomes	ACC deaminase gene	ET	Bacteria with ACC deaminase enzymes play a role in drought tolerance by degrading ethylene and influencing stress-related gene expression in plants.	[43]

## Data Availability

Not applicable.
